# The Application of Latent Class Analysis for Investigating Population Child Mental Health: A Systematic Review

**DOI:** 10.3389/fpsyg.2019.01214

**Published:** 2019-05-29

**Authors:** Kimberly J. Petersen, Pamela Qualter, Neil Humphrey

**Affiliations:** Manchester Institute of Education, University of Manchester, Manchester, United Kingdom

**Keywords:** latent class analysis, latent profile analysis, LCA, LPA, mental health, child, systematic review

## Abstract

**Background:** Latent class analysis (LCA) can be used to identify subgroups of children with similar patterns of mental health symptoms and/or strengths. The method is becoming more commonly used in child mental health research, but there are reservations about the replicability, reliability, and validity of findings.

**Objective:** A systematic literature review was conducted to investigate the extent to which LCA has been used to study population mental health in children, and whether replicable, reliable and valid findings have been demonstrated.

**Methods:** Preferred Reporting Items for Systematic Reviews and Meta-Analyses (PRISMA) guidelines were followed. A search of literature, published between January 1998 and December 2017, was carried out using MEDLINE, EMBASE, PsycInfo, Scopus, ERIC, ASSIA, and Google Scholar. A total of 2,748 studies were initially identified, of which 23 were eligible for review. The review examined the methods which studies had used to choose the number of mental health classes, the classes that they found, and whether there was evidence for the validity and reliability of the classes.

**Results:** Reviewed studies used LCA to investigate both disparate mental health symptoms, and those associated with specific disorders. The corpus of studies using similar indicators was small. Differences in the criteria used to select the final LCA model were found between studies. All studies found meaningful or useful subgroups, but there were differences in the extent to which the validity and reliability of classes were explicitly demonstrated.

**Conclusions** : LCA is a useful tool for studying and classifying child mental health at the population level. Recommendations are made to improve the application and reporting of LCA and to increase confidence in findings in the future, including use of a range of indices and criteria when enumerating classes, clear reporting of methods for replicability, and making efforts to establish the validity and reliability of identified classes.

## Introduction

### Rationale

It is important for researchers in the field of child mental health to be able to identify subgroups of children, within the general population, who have similar patterns of mental health symptoms and/or strengths. These subgroups can be studied further to investigate matters such as which mental health classes are commonly found in the general population, how prevalent they are, what causes them, what future outcomes they predict, and whether mental health classes change over time. In addition, identifying these subgroups is important for practitioners in the field because it allows a targeted approach to mental health promotion.

An increasingly popular method for identifying subgroups is latent class analysis (LCA). LCA is a cross-sectional latent variable mixture modeling approach. Like all latent variable mixture modeling approaches, LCA aims to find heterogeneity within the population. It does this by analyzing individuals' patterns of behavior, such as mental health indicators, and finding common types, called classes (Collins and Lanza, 2010). Each individual is probabilistically assigned to a class. That results in subgroups of individuals, who are most similar to each other and most distinct from those in other classes (Berlin et al., 2013). LCA can be carried out with categorical and/or continuous indicators, although LCA with continuous indicators is often called latent profile analysis (Muthen and Muthen, 1998-2017). For simplicity, here the term LCA is used for both analyses.

LCA has many strengths over other methods that can also be used to identify subgroups of children with similar patterns of mental health indicators. For example, children are often classified into groups according to specific mental health diagnoses on DSM-5 (American Psychiatric Association, 2013) or by exceeding a cut-off point on a continuous mental health measure (e.g., clinical cut-off points on the Strengths and Difficulties Questionnaire; Goodman, 2001). However, in both cases, individuals either side of the threshold for classification are very similar. Furthermore, heterogeneity within subgroups could be substantial (Dowdy and Kamphaus, 2007). Because those methods result in artificial, indistinct subgroups, any associations between them and other factors will be attenuated or spurious (MacCallum et al., 2002). LCA, on the other hand, uses maximum likelihood estimation to form subgroups which are internally homogenous and externally heterogeneous (Berlin et al., 2013). Therefore, researchers can more confidently use those subgroups to investigate relationships with other salient constructs, such as risk or promotive factors.

Another strength of LCA is that it is a model-based technique. A key advantage of model-based techniques over heuristic cluster techniques (e.g., k-means clustering) is that they provide fit statistics. Fit statistics assist researchers in choosing the most appropriate model for the data (Vermunt and Magidson, 2002), and can be used to compare models for hypothesis testing (Miettunen et al., 2016). Furthermore, LCA provides information on the probability that an individual is within a particular class (Vermunt and Magidson, 2002) and, as models can be extended to include covariates, this classification information can be retained in the broader model so measurement error can be accounted for (Muthen and Muthen, 1998-2017; DiStefano and Kamphaus, 2006). Thus, researchers have more flexibility and accuracy when studying mental health subtypes and associated factors.

Despite those potential benefits, critics have raised major concerns about the application of latent variable mixture modeling techniques like LCA (Bauer and Curran, 2003; Lenzenweger, 2004; Sterba and Bauer, 2010). First, LCA is usually conducted in an exploratory manner, whereby an increasing number of classes are fitted to the data and the best fitting model is chosen. The final class solution is decided upon by the researcher, who may use various criteria to choose the final model. Because this decision relies somewhat on the researchers judgement, results may not be replicable (van de Schoot et al., 2017). Second, LCA is a data-driven approach, meaning that identified classes could be statistical artifacts which lack validity and reliability (Bauer and Curran, 2004). Without establishing validity and reliability of classes, it is difficult to infer whether the classes represents naturally occurring subgroups in the population or whether they are sample specific.

There are ways to address some of the issues raised above. For example, researchers can be transparent about the methods used to derive classes and the decisions made, so that findings can be critically appraised and replicated (Collins and Lanza, 2010; van de Schoot et al., 2017). In addition, identified classes can be validated by investigating whether there are expected relationships between classes and other variables (Lenzenweger, 2004; Collins and Lanza, 2010; Hicks et al., 2017). Reliability of classes can be tested by conducting the same analysis in different samples, or with a subset of the same sample, to see if they are consistently found (Bauer and Curran, 2004; Lenzenweger, 2004). Studies may not always apply that level of rigor to their analyses.

Despite such concerns, LCA is being used more widely. This is partly due to the availability of software and increased computational capacity, which are required to carry out the analysis (Miettunen et al., 2016). In addition, a number of researchers have published papers encouraging the use of LCA in mental health and developmental research because it is well suited to addressing pertinent questions in the field. For example, Lanza and Cooper (2016) encourage the application of LCA for studying complex multidimensional phenomena, such as mental health, because multiple aspects of individual functioning can be studied holistically. Other researchers have suggested that LCA is an important analytic tool for studying developmental heterogeneity in the population (von Eye and Bergman, 2003; Scotto Rosato and Baer, 2012; Berlin et al., 2013). In addition, it can be used to assess the impact of universal mental health interventions on groups with similar mental health typologies (Greenberg and Abenavoli, 2017). LCA, then, has utility in the field of child mental health and may continue to grow in popularity as a statistical tool for researchers and practitioners.

### Objectives

Despite the potential utility of LCA in the field, to date, there has been no research synthesis of studies which have used this method to investigate patterns of mental health symptoms and/or strengths in children in the general population. Therefore, it is not known to what extent LCA has been used for this purpose, whether there is a corpus of studies which produced similar findings, whether models are selected using appropriate criteria, or whether theoretically or practically meaningful mental health subtypes have been identified.

The current systematic review was carried out to provide an overview of research which has used LCA to study subgroups of children with similar patterns of mental health symptoms and/or strengths. In particular, the review focused on children in the general population, who are aged between 4 and 11 years. The age range was restricted in order to allow comparability between studies. Comparing the mental health of very young children to older children is problematic because behavior that are considered developmentally appropriate for a very young child, (e.g., hitting out or tantrums) are symptoms of externalizing problems in older children (Campbell et al., 2000; Carter et al., 2004; Fanti and Henrich, 2010). Similarly, mental health among adolescents may be qualitatively different from that of younger children. Adolescence is an important period of physical, social, and emotional change, which may impact an individual's mental health. In addition, a number of specific mental health problems become more prevalent in adolescence, such as alcohol and drug abuse, risky sexual behaviors, criminal activity, eating disorders, and self-harm (Moffitt, 1993; Eccles et al., 1996). That means comparing adolescent and child mental health cannot be done on a like-for-like basis. In addition, only studies including samples that were approximately representative of the general population were included. Studies that target specific mental health or “at risk” groups may identify mental health classes which are not commonly seen in the general population, making a comparison between findings difficult.

The main aim of the review was to investigate which aspects of mental health have been studied using LCA, and whether results were comparable. In addition, the review aimed to compare the methods used to select the final class models across studies, and investigate the extent to which classes were shown to be valid and reliable. This is important in order to be able to provide researchers and practitioners in the field of child mental health with (a) an appraisal of the current applications and potential utility of LCA for studying population mental health, (b) an indication of the rigor with which the method is currently applied, including whether the validity and reliability of found classes are demonstrated, and (c) recommendations for improving the way in which LCA is used in this area.

### Research Questions

1. Which aspects of population child mental health have been studied using latent class analysis and is there a corpus of comparable results?
2. What methods have been used to decide on the final number of mental health classes?
3. To what extent are found classes shown to be valid and reliable?

## Methods

The methodology and inclusion/exclusion criteria were specified in advance and documented in a protocol (https://www.crd.york.ac.uk/prospero/ Reference: CRD42017083749).

### Eligibility Criteria

Studies Were Included in the Review if They Met the Following Criteria:

- Participants were aged between 4 and 11 years (inclusive).
- A general population sample was used, i.e., the sample was not targeted at or “enriched” for a specific group, such as those with a mental health diagnosis or those identified as being at high risk for developing mental health difficulties.
- Cross-sectional latent class analysis was used with categorical or continuous indicators. Studies that used longitudinal data to create the classes, for example, studies that used latent class growth analysis to create classes of children with specific mental health trajectories, were not included. That was because the longitudinal nature of the data adds another layer of meaning and it would make it difficult to compare results between studies with different designs. However, studies were not excluded purely on the basis of including longitudinal data, for example, studies with distal outcomes were included, as were studies that had carried out latent transition analysis (a longitudinal extension to latent class analysis), providing they reported latent class analyses for each time point separately.
- The LCA analysis used mental health indicators only—mental health indicators could include mental health symptoms and/or subjective wellbeing, as set out in the dual-factor model of mental health (Greenspoon and Saklofske, 2001; Suldo and Shaffer, 2008; Antaramian et al., 2010). If non-mental health indicators were included in the latent class analysis, the study was not included because this would influence the formation of the classes (Berlin et al., 2013) and would not produce subtypes based purely on mental health. Therefore, it would make it impossible to compare study results on a like-for- like basis.

Studies were excluded if eligibility criteria were not met, if the article was not available in English, or if methods and results were not reported in sufficient detail to assess eligibility and compare studies. As a minimum, studies were required to report the sample age and population from which it was drawn, the mental health indicators used to derive classes, the statistical method(s) employed, the procedure followed for deriving classes, and the name and number of derived classes.

### Search Strategy

The search strategy included terms relating to (a) children, (b) latent class or latent profile analysis, and (c) mental health. The search terms were adapted for use with each bibliographic database and their thesaurus and mapping functions (see Supplementary Material S1 for full search terms).

### Data Sources

The following electronic bibliographic databases were searched: MEDLINE, EMBASE, PsycINFO, Scopus, ERIC, and ASSIA. In addition, Google Scholar was searched, along with the reference lists of eligible studies and review articles.

To reduce publication bias, research was considered from a variety of sources, including peer reviewed journals, unpublished research, conference papers, and doctoral theses. The database Scopus was chosen because it includes gray literature, and Google Scholar was searched because of its inclusion of a wide range of sources (Haddaway et al., 2015).

### Study Selection

Results from the initial searches were saved to Endnote and duplicates were removed. The first author scanned the title and abstract of all remaining records to identify studies that potentially met the inclusion criteria. The second author checked 20% of those to ensure consistency in sorting. The full-text versions of the remaining papers were assessed against the full inclusion and exclusion criteria by the first two authors. Disagreements or ambiguities were resolved through discussion with all authors.

### Data Extraction

A data extraction sheet was developed based on the review aims^1^. Information on sample characteristics, method, and results for each reviewed study were extracted. Study authors were contacted for further information when a potentially eligible study was found, but not enough information was provided for review (e.g., conference abstracts, unpublished thesis; *n* = 13).

### Data Analysis

Data on the aspects of mental health investigated using LCA, the method used for class selection, the findings and evidence for class validity or reliability were compared using summary tables.

### Quality Analysis

An adapted version of the *Guidelines for Reporting on Latent Trajectory Studies (GRoLTS) checklist* (van de Schoot et al., 2017) was used to perform quality assessments for the included studies. The adapted checklist contained 15 yes/no items, such as, “Is the software mentioned,” “Is entropy reported?,” “Are plots/bar charts included with the response patterns of the classes/profiles in the final solution.”^2^ This checklist was important because clear and transparent reporting is required to be able to interpret and critically appraise results, and draw comparisons between studies.

## Results

### Study Selection and Characteristics

**Figure 1:**
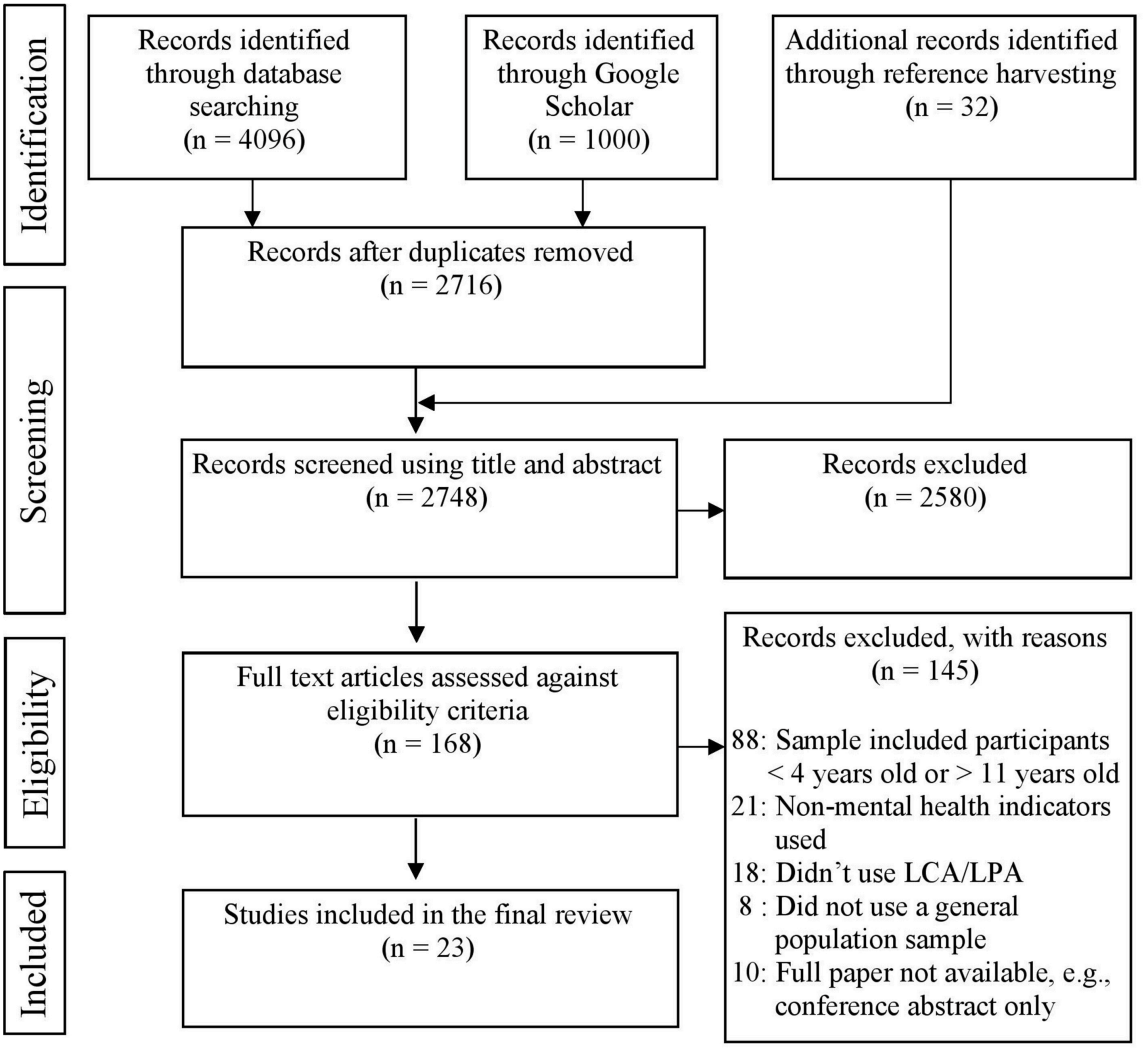
PRISMA flow diagram illustrating the flow of information at each stage of the review process.

The review process identified 23 eligible studies from the 2,748 studies gathered through the initial searches (see Figure 1 for PRISMA flowchart of studies retained and excluded at each stage of the review process). Within these studies, 97 eligible analyses were carried out. Eleven studies also carried out other analyses which did not meet the criteria for review (see Table 1).

**Table 1:** Key characteristics of included studies, sorted by aspect of mental health studied.

**Authors; sample**	Analyses (^*^non-eligible)	**MH indicators (measure)**	**Software: selection criteria**	**Actual classes (percent/proportion per class)**	**Evidence for validity of classes**	**Evidence for reliability of classes**
**INTERNALIZING PROBLEMS: ANXIETY**						
(Carey et al., 2017) University of Cambridge study(*n* = 817)aged 8–9 years	1 ^*^1 (> 11 years old)	General anxiety; test anxiety; math anxiety; 3 subscales (RCMAS-2: S; CTAS)	Mplus v7.11 LMR-LRT; BLRT; AIC; BIC; entropy; checked for spurious classes; parsimony	1 high anxiety (3.3%); 2 moderate anxiety (21.3%); 3 slight anxiety (38.8%); 4 low anxiety (36.6%)	differential relationships; group differences; developmental differences	Informal test; different for adolescents
(Ferdinand et al., 2006) Zuid Holland study(*n* = 203) Aged 8–11 years	1 ^*^3 (1 >11 years old; 2 referred groups)	Social anxiety; separation anxiety; 18 items (MASC- Dutch version)	Mplus v3.0 BIC; LMR-LRT	1 high/moderate problem levels (72.8%); 2 low problem levels (27.4%)	Group differences	Invariance test; different for some ages/referred samples
**INTERNALIZING: ANXIETY AND DEPRESSION**						
(Wadsworth et al., 2001)CBCL 4-18 validation studies (*n* = 1,076)Aged 4–11 years	2 ^*^3 (1 > 11 years old; 2 referred)	Depression; anxiety; 14 items (CBCL)	Program written by Eaves and modified by Hudziak et al. (1998) Likelihood ratio chi-square test; clinically informative classes only	**Girls:** 1 low (0.51); 2 mild (0.39); 3 moderate depression and anxiety (0.09). **Boys:** 1 low (0.46); 2 mild (0.46); 3 moderate depression and anxiety (0.08)	Group differences	Informal test; similar across ages and sexes; no low symptom group in referred sample
**INTERNALIZING: OBSESSIVE COMPULSIVE**						
(Althoff et al., 2009) The Netherlands Twin Registry(*n* = 10,194) aged 7 years;(*n* = 6,448) aged 10 years	4 ^*^3 (>11 years old)	Obsessive compulsive behavior; 8 items (CBCL)	Latent Gold 4.0 BIC; ssaBIC; face validity	**Age 7 boys:** 1 no symptoms (0.83); 2 worries and has to be perfect (0.10); 3 thought problems (0.07); 4 OCS (0.01). **Age 7 girls:** 1 no symptoms (0.79); 2 worries and has to be perfect (0.16); 3 thought problems (0.03); 4 OCS (0.01). **Age 10 boys:** 1 no symptoms 2487(0.80); 2 worries and has to be perfect (0.11); 3 thought problems (0.80); 4 OCS (0.01). **Age 10 girls:** 1 no symptoms (0.80): 2 worries and has to be perfect (0.16); 3 thought problems (0.04); 4 OCS (0.02).	Heritable; identified ODD domains	Informal test; similar across different ages and sexes
**EXTERNALIZING: ADH**						
(Althoff et al., 2006) The Netherlands Twin Registry(*n* = 1,219 parent-parent analysis; *n* = 598 parent-teacher analysis)Aged 10 years	6	Cognitive problems/inattention; ADHD Index; 21 parent and 22 teacher items(CPRS-R:S; CTRS-R:S)	LCAP BIC	**Mother/male:** 1 no or mild symptoms (60.8%); 2 mild inattentive (12.9%); 3 severe inattentive (12.0%); 4 hyperactive-impulsive (6.6%); 5 severe combined (7.7%). **Mother/female:** 1 no or mild symptoms (74.5%); 2 mild combined (15.6%); 3 severe inattentive (5.7%); 4 severe combined (4.1%). **Father/male:** 1 no or mild symptoms (69.7%); 2 mild inattentive (19.5); 3 severe combined (10.8%). **Father/female:** 1 no or mild symptoms (76.1%); 2 mild combined (17.5%); 3 severe inattentive (6.4%). **Teacher/males:** 1 no or mild symptoms (68.7%); 2 severe inattentive (13.8%); 3 hyperactive-impulsive (9.3%); 4 severe combined (8.2%). **Teacher/females:** 1 no or mild symptoms (75.3%); 2 mild combined (18.5%); 3 severe inattentive (6.2%).	Group differences; identified ADHD domains	Informal test; rater and sex differences
(Hudziak et al., 1999) CBCL 4-18 validation studies (*n* = 1,076)Aged 4–11 years	1 ^*^3 (1 > 11 years old; 2 referred samples)	Attention problems; 11 items (CBCL)	Program written by Eaves and modified by Hudziak et al. (1998) Likelihood ratio chi-squared test. clinically uninformative classes, e.g., <1% of the sample were excluded	**Age 4–11 girls: 1** low/none (0.59); 2 mild (0.25); 3 moderate attention problems (0.16). **Age 4–11 boys:** 1 low/none (0.42); 2 mild (0.44); 3 moderate attention problems (0.14)	Group differences	Informal test; similar across ages, sexes, referred and non-referred groups
(Romano et al., 2002) NLSCY (*n* = 822–968 per analysis)Aged 4, 5, 6, 7, 8, 9, 10, 11 years	32 ^*^4 (<4 years old)	Analysis 1: hyperactivity- impulsivity; 3 items Analysis 2: inattention; 3 items (NLSCY interviews items)	LEM χ2; likelihood ratio chi-square test; The Cressie-Read goodness-of-fit statistic; AIC; BIC; % variance explained	**Hyperactive-impulsive: Girls:** 1 low (0.37–0.75); 2 medium (0.15–0.46); 3 high symptoms (0.09–0.17). **Boys:** 1 low (0.38–0.65); 2 medium (0.23–0.46); 3 high symptoms (0.09–0.23). **Inattentive: Girls:** 1 low (0.63–0.72); 2 med (0.24–0.35); 3 high symptoms (0.01–0.10). **Boys:** 1 low (0.45–0.62); 2 med (0.28–0.54); 3 high symptoms (0.01–0.18)	Group differences	Informal test; similar across ages and sexes
**EXTERNALIZING: AGGRESSION**						
(Baillargeon et al., 1999) NLSCY(*n* = 822–968 each analysis)Aged 4, 5, 6, 7, 8, 9, 10, and 11 years	16 ^*^4 (<4 years old)	Physical aggression; 3 items (NLSCY interview items)	Pre-release command line version of MLLSA for CDAS 4.0 χ2; likelihood-ratio chi- square test; % variance explained by model; % correctly allocated	Low, medium and high aggression classes for all, except 6-year old boys: **Boys:** 1 low (0.73–0.82); 2 medium (0.17–0.20); 3 high aggression (0.02–0.06). **Girls:** 1 low (0.77–0.90); 2 med (0.10–0.22), 3 high aggression (0.00–0.01)	Group differences	Formal test; similar structure across ages and sexes (except 6 year-old boys)
(Lee et al., 2007) NLSCY(*n* = 12292; ~900 per analysis)Aged 5, 6, 7, 8, 9, 10, and 11 years	14	As Baillargeon et al. (1999)	LEM χ2; likelihood ratio chi -square test; BIC; % variance explained	**Boys each age:** 1 low (0.78); 2 medium (0.18); 3 high aggression (0.04). **Girls each age:** 1 low (0.83–0.91); 2 medium (0.09–0.17); 3 high aggression (0.01–0.02)	Group differences	Informal test; similar across ages and sexes
**EXTERNALIZING: OPPOSITIONAL DEFIANT BEHAVIOR**						
(Kuny et al., 2013) The Netherlands Twin Registry(*n* = 7,597) aged 7 years; (*n* = 6,548) aged 10 years	2 ^*^1 (> 11 years old)	oppositional behavior; 6 items (CPRS-R:S)	Latent Gold BIC; AIC; entropy; classification error; bootstrapped p-value; average bivariate residuals	**Age 7:** 1 no symptoms (0.70); 2 defiant (0.12); 3 irritable (0.11); 4 high symptoms (0.08). **Age 10:** 1 no symptoms (0.69); 2 defiant (0.12); 3 irritable (0.10); 4 high symptoms (0.09)	Differential relationships; group differences; heritable; identified domains of ODD	Informal test; similar across ages
**EXTERNALIZING: BROAD**						
(van Lier et al., 2003b) Intervention study, the Netherlands (*n* = 636) Mean age 6.9 years (SD 0.6)	1	Conduct problems; ODD problems; ADH problems; 19 items (CBCL/4–18)	Mplus 2.02 BIC; Bayes factor	Model without covariates: 1 intermediate conduct problems and high ADH and ODD problems (21%); 2 intermediate probabilities for ODD problems and ADH but low conduct problems (49%); 3 low problems for all (30%)	Differential relationships; identified disruptive behavior syndromes	
(van Lier et al., 2003a)Intervention study, the Netherlands (*n* = 622 at T1, *n* = 560 at T2)Mean age T1 6.9 years (SD 0.6); **T2:** 1 year later	2	^*^as van Lier et al., 2003b	Mplus 2.02 BIC; Bayes factor	**T1:** 1 high ODD and ADH problems and intermediate conduct problems (16%); 2 no conduct problems and intermediate ODD and ADH problems (46%); 3 low problems (28%). **T2:** 1 high ODD problems and ADH problems and intermediate conduct problems (19%); 2 no conduct problems and intermediate ODD and ADH problems (48%); 3 low problems (33%)	Differential relationships; group differences	Informal test; similar across time
**EXTERNALIZING: SITUATION**						
(Fergusson et al., 2009) Christchurch Health and Development Study (*n* = 1,046)Aged 7–9 years	1	Conduct problems (6 items) (Connors' questionnaire; Rutter questionnaire)	PANMARK BIC; log likelihood ratio chi-square test	1 no problems (83.2%); 2 mother-reported problems (5.8%); 3 teacher reported problems (7.6%); 4 generalized problems (3.4%)	Differential relationships	
(Sulik et al., 2017) The Family Life Project (*n* = 960–1086)Aged 5 and 7 years	2	Conduct problems (5 teacher-rated and 5 parent-rated items) (SDQ)	Mplus 7.3 BIC; LMR-LRT; BLRT; interpretability; theoretical appropriateness; parsimony; class separation	**T1:** 1 low cross-context (55%); 2 home context (25%); 3 school context (12%); 4 high cross-context (8%). **T2:** 1 low cross-context (55%) 2 home context (20%); school context (*n* = 184, 16%); 4 high cross-context (8%)	Differential relationships; development differences	Invariance test; partial invariance across time
**MULTIPLE ASPECTS**						
(Basten et al., 2013)Generation R dataset (*n* = 6131)Aged 5–7 years	1	Emotionally reactive; anxious/depressed; somatic complaints; withdrawn; attention problems; aggressive behavior; 6 subscales (CBCL/1.5–5)	Mplus v6 BIC; BLRT; entropy; class differentiation	1 highly problematic (1.8%); 2 internalizing (5.3%); 3 externalizing/emotionally-reactive (7.3%); 4 no problems (85.6%)	Differential relationships; group differences; evidence for symptom comorbidity	Similar to those found in other studies
(Basten et al., 2016)^**^as (Basten et al., 2013)	1 ^*^2 (<4 years old)	as (Basten et al., 2013)	^**^as (Basten et al., 2013)	^**^as (Basten et al., 2013)	Developmental differences	Invariance test; different with very young children
(Bradshaw et al., 2015) RCT universal school program effectiveness trial (*n* = 12,334) Aged 6–7 years	1	Concentration problems; aggressive and disruptive behaviors; positive behaviors; emotion regulation problems; 4 subscales (TOCA-C)	Mplus 6.1 AIC; BIC; ssaBIC; LMR-LRT;	1 socially-emotionally skilled (33.6%); 2 normative (36.5%); 3 at risk (23.3%); 4 high-risk (6.6%)	Differential relationships	
(McElroy et al., 2017) Avon Longitudinal Study of Parents and Children(*n* = 4,525) Aged7.5 years	1 ^*^1 (> 11 years old)	Presence of psychiatric disorder; 8 items (DAWBA)	Programme not reported AIC; BIC; ssaBIC; LMR-LRT; entropy	1 high risk/multimorbid (1.5%); 2 low endorsement/normative (79.9%); 3 externalizing (2.5%); 4 internalizing (16.1%)	Developmental differences; identified comorbid symptoms	Invariance test; similar across ages
(Nozadi et al., 2016) Adaption study in mid-Atlantic USA (*n* = 189)Aged 10 years	1	Internalizing problems; externalizing problems; attention problems; anxiety-parent report; anxiety- child report; presence of disorder; 4 subscales/items (K-SADS; CBCL; SCARED)	Mplus 5 BIC; BLRT; interpretability	1 healthy (59%); 2 anxiety (25%); 3 attention anxiety (12%); 4 severe ADHD class (3%)	Differential relationships	
**MULTIPLE ASPECTS CONT**.						
(Racz et al., 2015) school prevention models study mid-Atlantic USA(*n* = 2,814)T1 aged 5–6 years; T2 aged 6–7 years	2	Concentration problems; prosocial behavior; disruptive behavior; emotion problems; 4 subscales (TOCA-C)	Mplus 7 BIC; ssaBIC; log-likelihood ratio chi square test; LMR-LRT; class size; whether classes added any descriptive or unique information	**T1:** 1 well adapted (49.7%); 2 concentration problems (37.5%); 3 at-risk T1 (12.8%). **T2:** 1 well adapted (40.2%); 2 concentration problems (40.5%); 3 at-risk (19.4%)	Differential relationships; group differences; differential development	Invariance test- partial invariance; similar across time points
(Vendlinski et al., 2014) Wisconsin Twin Project (*n* = 1,578 boys, n = 1,645 girls)Mean age 7.5 years (SD = 0.92)	2	Depression; over anxiousness; separation anxiety; conduct problems; oppositional defiant problems; inattention; 55 items (HBQ)	LatentGOLD BIC; Boot strapped p-value for chi-square test	**Girls:** 1 mildly anxious (0.16); 2 moderately oppositional (0.15); 3 moderately impulsive and attentive (0.14); 4 moderately anxious (0.12); 5 low symptom (0.11); 6 mildly oppositional and impulsive (0.11); 7 moderately anxious and non-conduct externalizing (0.09); 8 moderately externalizing (.08); 9 moderately internalizing and severely externalizing (0.04). **Boys:** 1 low symptom (0.17); 2 mildly oppositional and impulsive (0.17); 3 moderately impulsive and inattentive (0.13); 4 mildly anxious (0.13); 5 moderately oppositional (0.11); 6 moderately anxious and non-conduct externalizing (0.10); 7 moderately externalizing (0.08); 8 severely impulsive and inattentive (0.06), 9 moderately internalizing and severely externalizing (0.05)	Differential relationships; group differences	Informal test; similar across ages and sexes
**TWO DISTINCT ASPECTS**						
(H. I. Lanza, 2011) The National Institute of Child Health and Human Development (*n* = 1,072)Aged 9–10 and 10–11 years	2 ^*^ 1 (> 11 years old)	ODD symptoms; depression; 14 items (DBD Ratings Scale; CBCL; DSM-IV Affective Problems Scale)	PROC LCA BIC; AIC; interpretability; conceptual implications; parsimony	**4th Grade:** 1 very low ODD and depressive symptoms (0.50); 2 low ODD (0.39); 3 moderate ODD and low depressive symptoms (0.11). **5th Grade:** 1 very low ODD and depressive symptoms (0.46); 2 low ODD (0.43); 3 moderate ODD and low depressive symptoms (0.12)	Differential relationships identified some comorbidity of disorders	Informal test; similar across age
(Wall et al., 2016)Sample of families in Cyprus (*n* = 1,366 families)Aged 7–11 years	1	Callous-unemotional traits; conduct problems (2 subscales) (ICU-P; Child Symptom Inventory for Parents-4; CSI-4-P)	Mplus 6.1 BIC; AIC; LMT-LRT; entropy; mean posterior probability scores	1 low risk (67.2%); 2 high conduct problems/ low callous-unemotional traits (7.9%); 3 moderate conduct problems/ callous-unemotional traits (8.4%); 4 high callous-unemotional traits (9.4%); 5 high conduct problems/ callous-unemotional traits (7.2%)	Differential relationships; group differences	

*ADHD, Attention deficit disorder; AIC, Akaike information criterion; BIC, Bayesian information criterion; BLRT, bootstrap likelihood ratio test; CDAS, categorical data analysis; CBCL, Child Behavior Checklist; CPRS-R:S, Connors' Parent Rating Sale-Revised: short from; CTRS-R:S, Connors' Teacher Rating Scale-Revised: short form; CSI-4-P, Child Symptom Inventory for Parents-4; CTAS, The Children's Test Anxiety Scale; DAWBA, Development and Wellbeing Assessment; DBD, Disruptive Behavior Disorders; HBQ, The MacArthur Health and Behavior Questionnaire; ICU-P, Inventory of Callous-Unemotional Traits- parent report; K-SADS, Kiddie-Schedule for Affective Disorders and Schizophrenia for School-Age Children; LCA, latent class analysis; LCAP, LCA program; LEM, Log-linear and event history analysis with missing data using the EM algorithm; LMR-LRT, Lo-Mendel–Rubin likelihood ratio test; LPA, latent profile analysis; MLLSA, maximum-likelihood latent-structure analysis; MASC, Multidimensional Anxiety Scale for Children; NLSCY, National Longitudinal Survey of Children and Youth; OCS, obsessive compulsive symptoms; ODD, oppositional defiant disorder; RCMAS-2:S, The Revised Children's Manifest Anxiety Scale: Second Edition: short form; SCARED, Screen for Child Anxiety Related Disorder Questionnaire; SDQ, The Strengths and Difficulties Questionnaire; ssaBIC, sample size adjusted BIC; TOCA-C, Teacher Observation of Classroom Adaptation–Checklist*.

*χ^2^ = Pearson's chi-squared test*.

Samples were drawn from a range of sources, including; large national databases, such as the Netherlands Twin Registry; large community wide research projects, such as The Family Life Project; or from datasets obtained through other research projects (see Table 1). Three pairs of studies used identical or very similar samples and the same mental health indicators for one of their latent class analyses (Baillargeon et al., 1999 and Lee et al., 2007; Basten et al., 2013 and 2016; van Lier et al., 2003a and 2003b). Sample sizes ranged between 189 and 12,334. Fifteen studies examined mental health classes in mixed sex samples and eight examined mental health classes for boys and girls separately.

### Quality Analysis Results

The full quality of reporting and replicability assessment against relevant GRoLTS criteria is presented in the Supplementary Material S4. No studies were excluded from the review on the basis of reporting quality. However, the results highlight areas which are inconsistently or poorly reported, including; entropy values, the number of random start values and final iterations, and plots for all class solutions.

### Individual Study Characteristics and Results

Individual study characteristics, such as sample, LCA method, identified classes and evidence for validity and/or reliability of classes are presented in Table 1.

### Synthesized Findings

#### Comparison of Study Findings

All reviewed studies used indicators of psychopathology, but some chose indicators specific to a particular symptom (e.g., physical aggression), a specific disorder (e.g., attention deficit hyperactivity disorder; ADHD), or a broadband view of mental health which included internalizing and externalizing symptoms (see Table 1 for studies ordered according to aspects of mental health they examined). Only two studies considered positive aspects of behavior alongside symptoms (Bradshaw et al., 2015; Racz et al., 2015); no studies considered subjective wellbeing. General comparisons between studies looking at similar aspects of mental health are discussed.

##### Studies Using Internalizing Symptoms as Indicators

Both studies that used LCA to investigate anxiety types found ordinal classes of anxiety, despite examining different forms of anxiety and using different indicators. They also found that the low symptom class was *not* the largest group. Wadsworth et al. (2001) similarly found ordinal classes when investigating symptoms of depression and anxiety, and low and mild symptom groups were approximately equal in size. Indicators of another internalizing disorder, however, resulted in qualitatively different classes. Althoff et al. (2009) examined classes of obsessive-compulsive symptoms in four different samples and found that four classes consistently emerged; a large no symptom class, a high symptom class, and two other classes which represented specific symptom types.

##### Studies Using Externalizing Behaviors as Indicators

Of all the LCA analyses that investigated patterns of externalizing symptoms, 72 out of 77 (93%) found the non-symptomatic class to be the largest.

Studies that used indicators for a specific aspect of mental health, such as hyperactivity, inattention, or physical aggression, found classes that differed in symptom severity alone (Baillargeon et al., 1999; Hudziak et al., 1999; Romano et al., 2002; Lee et al., 2007). When indicators were broader and covered a particular disorder, such as attention deficit disorder, oppositional defiant behavior, or a broad range of behavior problems, classes differed in both type and severity (van Lier et al., 2003a,b; Althoff et al., 2006; Kuny et al., 2013).

Additionally, two studies examined conduct problems in the home and school context by combining responses from parents and teachers. One carried out two analyses with the same sample at different time points (Sulik et al., 2017). All analyses identified subgroups of children with situation specific conduct problems—home or school. There was also a large group of children with no conduct problems, and a small group with generalized conduct problems.

##### Studies Using a Broad Range of Mental Health Indicators

Table 1 shows that seven studies focused on multiple aspects of mental health, spanning both internalizing and externalizing problems, with two also including indicators of positive behavior (Bradshaw et al., 2015; Racz et al., 2015). Studies used different measures as indicators of mental health (see Table 1). Measures varied from non-clinical school behavior checklists, such as the Teacher Observation of Classroom Adaptation-Checklist, to more clinical measures, such as the Kiddie Schedule for Affective Disorders and Schizophrenia. Due to the range of indicators used, results were not directly comparable. However, all studies using a broad range of mental health indicators found that low symptom classes were the largest group, with the exception of Vendlinski et al. (2014), who found that the most prevalent class for girls was the mildly anxious class, and Racz et al. (2015), who found that the well-adapted and concentration problems groups were equally large in their one of their analysis.

Some studies reported similar classes to each other despite using different measures. McElroy et al. (2017) and Basten et al. (2013) found a low symptom class, a comorbid class, a pure internalizing class, and a pure externalizing class or externalizing/emotionally reactive class. Vendlinski et al. (2014) also found similar classes, with the addition of specific mental health problems classes referred to as “moderately anxious” and “moderately impulsive and inattentive.”

Bradshaw et al. (2015) was the only study to find a class that had better than average functioning, and the only multiple aspects of mental health study to find ordinal categories. All other studies found qualitative differences as well as differences in problem severity.

##### Studies Using Indicators for two Distinct Mental Health Problems

The remaining two studies looked at two distinct aspects of mental health. Lanza (2011) studied oppositional disorder symptoms and depression and Wall et al. (2016) studied callous-unemotional traits and conduct problems. Those studies were largely concerned with identifying subgroups of children with pure and comorbid symptoms. Both studies found small comorbid classes, and large no symptom classes, as well as classes which had higher symptoms in one disorder than the other.

##### General Patterns of Results Across Studies

LCA was used to identify subgroups of children with distinct patterns of mental health. The mental health indicators used in different studies varied in their specificity to a particular disorder and their severity (i.e., clinical measure or school measure). This influenced the types of classes found, although some general patterns emerged. For instance, subgroups of children with qualitatively different patterns of mental health symptoms were identified when a wide range of indicators were used. Subgroups that differed in symptom severity alone were identified when the range of symptoms used as indicators was narrow or for certain symptom types (i.e., anxiety and depression). Additionally, most analyses (89%) found the largest class was the low or no symptom class, which contained between 17% and 91% of the sample, depending on the indicators used and total number of classes found. Some comparable classes were found in studies that had used similar indicators, but the number of studies was small.

#### Comparison of Study Methodologies

Studies used a variety of programs to estimate models (see Table 1). Fit statistics were the main tool for model selection in all included studies. Bayesian Information criteria (BIC) was used in 20 studies (87%). Nineteen of these studies (83%) used additional criteria, such as Akaike Information Criteria (AIC), sample size adjusted BIC (ssaBIC) Bootstrap Likelihood Ratio Test (BLRT), and Bayes factor. Seven studies (30%) used likelihood ratio difference tests either instead of BIC or in conjunction with it. Sixteen studies (70%) reported using other criteria, such as entropy, interpretability of classes, and model parsimony, to select the best model.

#### Evidence for the Validity of Identified Classes

The external validity of classes was demonstrated when differential relationships with other factors were identified. Thirteen of the 23 studies (57%) examined whether covariates differentially predicted class membership. Of those, three found that the classes had different antecedents (Basten et al., 2013; Nozadi et al., 2016; Sulik et al., 2017), one found that classes differentially predicted other outcomes (Fergusson et al., 2009), and eight found cross-sectional associations (van Lier et al., 2003a,b; Lanza, 2011; Basten et al., 2013; Bradshaw et al., 2015; Racz et al., 2015; Wall et al., 2016; Carey et al., 2017). All studies found that classes had differential relationships with at least one covariate. Four studies also identified some covariates that did not have different associations with class (van Lier et al., 2003b; Lanza, 2011; Nozadi et al., 2016; Wall et al., 2016). Overall, the results from these 13 studies indicate that LCA produced externally valid classes.

The validity of classes was also indicated by the extent to which they showed practical or theoretical utility. Table 1 indicates whether studies found meaningful classes, by identifying classes which represented either heritable phenotypes or theorized mental health typologies. Studies that found theoretically and practically interesting classes representing subgroups of children who followed different developmental courses, are also indicated, as are those which identified mental health differences among observed groups of children such, as age or sex differences. Classes which had practical utility, in that they classified children into mutually exclusive groups, so that symptom prevalence in the population could be estimated, are also noted. Overall, each study in the review produced classes that served a practical or theoretical purpose, attesting to their validity as meaningful constructs.

#### Evidence for the Reliability of Identified Classes

Six studies (indicated in Table 1) reported that they had carried out formal statistical tests of measurement invariance to assess whether the same classes were found in different samples. Invariance or partial invariance was found in all cases apart from in one study (Baillargeon et al., 1999), which found that classes of aggression were non-invariant across gender- in other words, the classes were not structurally equivalent for boys and girls.

Ten studies (43%) made informal assessments of whether the same classes were found in different samples (see Table 1). Althoff et al. (2006) found different classes of ADHD behavior in boys compared to girls. However, the other nine studies concluded that classes were generally similar in different samples. Although not as robust as formal tests of measurement invariance, these studies suggest that similar classes can be found in different samples and show a degree of reliability. Only one study made explicit comparisons with the classes found in their study and those found using other samples (Sulik et al., 2017); the authors concluded that their class solution was consistent over time, and with other similar studies.

Overall, 16 out of the 23 studies (70%) carried out the analysis in more than one sample and reliability of classes is generally supported. Seven studies (30%) did not carry out additional analyses so the reliability of classes is unknown.

## Discussion

This systematic review identified 23 eligible studies, which conducted 97 LCA analyses of mental health indicators, for children in the general population. The review found that studies had used a range of mental health indicators to study mental health types but few had used the same indicators. Therefore, we did not find a large corpus of similar results for any area of population mental health. Comparing study methodologies and results revealed that there was variation in how the final number of classes were selected and the extent to which the validity or reliability of found classes was demonstrated.

### Overview of How LCA Has Been Used to Study Population Mental Health in Children

Each study in the review identified subgroups of children with particular patterns of mental health, thus capitalizing on the fact that LCA is a person-oriented method, which focuses on heterogeneity in the population. It is noteworthy that, even in this small sample of studies, LCA has been applied in different ways. For example, it has been used to classify children into distinct subgroups to investigate population prevalence of symptoms (e.g., Baillargeon et al., 1999), to determine whether subtypes of disorders emerged or whether the difference between children's symptoms was simply quantitative (e.g., Carey et al., 2017), to investigate symptoms expressed in a particular context (e.g., Sulik et al., 2017), and to investigate the comorbidity of distinct disorders (e.g., Wall et al., 2016). Once classes have been derived, these can be included in other analyses, for example to test the heritability of class types (e.g., Vendlinski et al., 2014), how they change over time (e.g., Basten et al., 2016), and what other factors predict them (van Lier et al., 2003a). It is clear that LCA is a useful and flexible statistical tool for researchers interested in researching population mental health in children.

All studies investigated symptoms of psychopathology in the general population and only two studies included some indicators of positive behavior (Bradshaw et al., 2015; Racz et al., 2015). Consequently, the majority of studies found that the no symptom class was the largest. This is consistent with evidence that most children in the general population do not experience mental health difficulties (Sadler et al., 2018). Therefore, it is surprising that 11% of studies found that the no symptom group was not the largest. In those studies, symptomatic groups may have been more prevalent because of cohort differences. It could also be because the measures tested a more normative set of feelings and behaviors. Providing an indication of average responses in the overall sample and for each class could help to interpret results like these better.

Focusing solely on mental health symptoms rather than positive mental health and wellbeing also means that heterogeneity was generally only observed in symptomatic groups. Research indicates that considering a wider range of symptoms *and* strengths captures a greater range of mental health heterogeneity (Rose et al., 2017; St Clair et al., 2017). Two of the reviewed studies examined a range of behavior from symptomatic to positive, leaving more opportunity for heterogeneity in none symptomatic children to be identified. One of those studies (Bradshaw et al., 2015), did, indeed, find a “better than average functioning” class, suggesting heterogeneity in the non-symptomatic groups. If researchers are interested in heterogeneity across the whole population rather than identifying symptomatic groups alone, LCA can be applied for that purpose. Proponents of the dual-factor model of mental health suggest that a focus on symptoms, at the expense of subjective or psychological wellbeing, fails to identify important groups, such as those that have few symptoms but low levels of wellbeing, or those with many symptoms but high levels of wellbeing (Greenspoon and Saklofske, 2001; Suldo and Shaffer, 2008; Antaramian et al., 2010; Lyons et al., 2012). Therefore, researchers interested in mental health development in general population samples may consider including a broad range of mental health indicators to capture more heterogeneity.

Rather than finding a corpus of studies using similar indicators to measure mental health, the review produced studies which had used an array of indicators. Thus, it is difficult to draw firm conclusions about what mental health classes exist in the population. However, it indicates areas where LCA has been used, and some patterns between findings have been identified. The review highlighted that when studies used indicators for a narrow aspect of mental health, they tended to find classes which differed in severity of symptoms alone. This is unsurprising, as distinct mental health patterns are unlikely to arise when symptoms are very similar in nature. The area of mental health being investigated may also influence what kinds of classes are found. For example, LCA studies that used a range of anxiety types, or anxiety and depression symptoms, found ordinal classes. This suggests that the level of anxiety and depression differentiates children more than the particular types of symptoms experienced. Because only a few studies have looked at this within the general population, further research using different indicators and samples would be needed to ascertain whether pure forms of anxiety or depression are commonly found in children.

In addition, studies that used a range of internalizing and externalizing problems as indicators, tended to find a large no symptoms class, a small multi-morbid class, and then some qualitatively different symptom classes, such as internalizing only, externalizing only, or a specific disorder. This suggests that similar classes are often observed in the general population. However, the particular indicators used can subtly alter the nature of the identified classes. It is, therefore, important for researchers to consider how the indicators used for latent class analysis may enable or restrict which classes are identified.

### Differences in Method of Class Selection

Studies included in the review often used different criteria to enumerate classes, reflecting the fact that there is no sole criterion for choosing the most appropriate latent class model (Nylund et al., 2007). The majority of studies in the review used BIC to test the relative fit of two models with different numbers of classes. Lower BIC values indicate better model fit, when parsimony is considered (Collins and Lanza, 2010). When conducting exploratory LCA, the best class solution is found by testing models of increasing numbers of classes and selecting the model with the lowest BIC. This is a useful method recommended by a number of researchers (e.g., Hagenaars and McCutcheon, 2002; Nylund et al., 2007), but, in cases where samples are large, relying on BIC alone can lead to the over extraction of classes (Specht et al., 2014). Three studies in this review (Baillargeon et al., 1999; Hudziak et al., 1999; Wadsworth et al., 2001) did not use BIC at all. Instead, they used chi-square statistics in order to choose between competing latent class models. Chi-square fit statistics test the absolute fit of the model, however, they are not always appropriate for testing different class models in LCA because, when data is sparse (i.e., when there are many possible response patterns with small frequencies) the chi-squared distribution may not be well approximated and, therefore, *p*-values can be unreliable. Parametric bootstrapping and posterior predictive checks can be used to overcome problems such as these (Nylund et al., 2007; Collins and Lanza, 2010; Masyn, 2013). Sixteen studies reported fit statistics in addition to BIC. Of these, nine reported additional information criteria such as AIC and ssaBIC. Research indicates the power of information criteria to detect the best solution depends on the degree of separation between classes, sample size and the number of indicators used, therefore, examining more than one statistic may be preferable (Tein et al., 2013). Other information criteria may also be used to help enumerate classes such as Consistent Akaike Information Criterion (CAIC) and Approximate Weight of Evidence (AWE) Criterion, although these were not applied in the studies included for review. All information criteria statistics indicate relative model fit when complexity of the model is taken in to account. Lower values indicate better fit, so class solutions with the lowest values are favored (Masyn, 2013; Nylund-Gibson and Choi, 2018). Nine studies also used likelihood-ratio tests (i.e., LMR-LRT and BLRT) to indicate whether adding a class significantly improved model fit. Studies have shown that these fit statistics provide useful additional information when choosing between different class models (Nylund et al., 2007; Tein et al., 2013). For both the LMR-LRT and BLRT, a significant p-value indicates that the model fit is significantly better for the k-class model, than the model with k-1 classes (Nylund et al., 2007). Two reviewed studies also applied Bayes factor to compare the fit of adjacent models. This is a Bayesian method which indicates the strength of support for a model compared to a model with one class more. Scores less than 3 indicate weak support and scores greater than 10 indicate strong support for the more parsimonious model (Nylund-Gibson and Choi, 2018). As research suggests that fit statistics have different strengths and provide different information, which can be used to aid the class enumeration, researchers should be encouraged to use and report multiple fit indices. This was not evident in all reviewed studies, and it is an area for improvement in future research.

While fit statistics may guide model selection, even the use of several fit indices cannot provide a definitive indication of the best model. Indeed, they may contradict each other. Therefore, after fit statistics have been used to narrow down the number of possible models, the substantive meaning of the classes should be considered in order to select the best class solution. For example, one might consider whether classes are in line with what would be expected from theory, whether they are easy they are to interpret, whether classes are large enough to be of interest to the researcher, and whether the solution is parsimonious (Collins and Lanza, 2010; Meeus et al., 2011; Masyn, 2013). Thirteen studies in this review (57%) did not report making any of these considerations when selecting the best class solution, suggesting that opportunities to select the most theoretically and practically meaningful classes might have been missed. Approximately a third of studies (30%) reported using classification quality information to select the best model, such as entropy and mean posterior probability scores. Other studies have also found that entropy is frequently used in the model enumeration process (Tein et al., 2013). Entropy is a measure of overall classification quality. Scores range from zero to one, with the higher value being the better value as far as classification is concerned. Whereas, mean posterior probability scores indicate the certainty to which individuals are assigned to each class. While classification information is useful for understanding the precision of latent class assignment, and should be reported, it does not indicate whether the model is a good fit to the data, and should not be used as a model selection tool (Masyn, 2013). Overall, the results of this review have highlighted that a range of fit indices and considerations about the substantive meaning of classes are being used for class selection in LCA in this specific field. Moving forward, researchers should consider whether they have used all the model selection tools available to them when choosing a final latent class solution.

Clear and detailed reporting of the methods is needed to allow replicability and the critical appraisal of results, because numerous decisions have to be made when selecting the final LCA model (van de Schoot et al., 2017). Quality analysis using the adapted GRoLTS checklist indicated that the majority of studies did report key information, such as the software used for the analysis, fit statistics used for model selection, and plots or charts of the final class solution. Other important aspects were inconsistently reported, such as entropy, and the total number of fitted models with fit indices. In addition, some aspects, which would be necessary for full transparency and to allow replicability, were poorly reported. For example, only a third of studies were clear as to whether they had applied parameter restrictions, and no studies provided the plots or bar charts for all class solutions, or made the syntax available as Supplementary Material. These findings are similar to assessments of the quality of reporting in latent trajectory studies (van de Schoot et al., 2017; see comparison figures included as Supplementary Material S4). Researchers may consider online Supplementary Material as an alternative avenue for displaying such information.

### Reliability and Validity of Mental Health Classes for Children in the General Population

In the review, few studies used the same indicators, making it difficult to draw comparisons between results. Where studies had used comparable indicators, some similarities emerged. For example, studies that looked at a broad range of internalizing and externalizing symptoms tended to find classes which represented internalizing, externalizing, and comorbid groups- something which is also found in the wider literature (Lilienfeld, 2003). Studies that looked at physical aggression found low, medium and high symptom groups. Additionally, studies which looked at parent and teacher reports of behavior found- no behavior problems high cross-context behavior problems, and context-specific behavior problem classes. Further research would need to be carried out to see if the same mental health classes are reliably found in different samples, especially where fewer studies have been carried out, such as with anxiety and depressive symptoms.

Many studies in the review demonstrated reliability in the classes they identified by finding the same classes in multiple analyses. However, none of the reviewed studies explicitly stated that the repeated analyses were conducted to test for reliability of classes, and the samples used for each analysis often differed in important ways. Additional analyses were mainly conducted to examine mental health differences in boys and girls or in different age groups. To establish confidence that classes are reliable and not sample specific, researchers should include formal tests of reliability with similar samples; this can be achieved by testing the model in more than one sample and carrying out tests to establish whether the classes are structurally similar, or by testing the model in half of the sample.

Validity of children's mental health classes were demonstrated in a number of ways in the reviewed studies. Each study showed that the classes were meaningful in some sense, however, most did not explicitly test the validity of classes by hypothesizing which classes should be identified and what relationship they would be expected to have with other factors. Identifying expected classes and expected relationships with other factors would indicate that the classes aligned with current theory and research as expected, and it would indicate that the classes were valid. Furthermore, when there is already a strong reason for assuming that there are subgroups of individuals with similar mental health symptoms or strengths, LCA can be applied as a confirmatory approach. By specifying the classes a priori, according to theory, and by setting parameter restrictions on the LCA model, more theoretically robust classes could be identified (Finch and Bronk, 2011; Schmiege et al., 2018). All reviewed studies used exploratory LCA, where different class models were generated and the best fitting model was chosen. Future studies should consider to what extent there is sufficient research evidence and theory for testing specific hypotheses about the classes that may be found. If so, formally testing them using confirmatory methods would be more appropriate.

### Strengths, Limitations, and Future Directions

Extensive searches of relevant databases, Google Scholar, and hand-searches were conducted. The search was limited to a 20-year period starting January 1997. Although some earlier studies may have been excluded, it was assumed that the majority of LCA studies in this area were conducted after 1997, since most software packages for LCA analyses were developed after this point (e.g., Muthen and Muthen, 1998-2017; Vermunt and Magidson, 2000; Lanza et al., 2007). A further limitation is that the review only included studies which examined the mental health of children aged between 4 and 11 years and those from the general population. As discussed previously, these restrictions were made in order to allow results to be compared on a like-for-like basis. Further research would need to be carried out to compare the results of studies that had used LCA to examine mental health in other groups.

**Figure 2:**
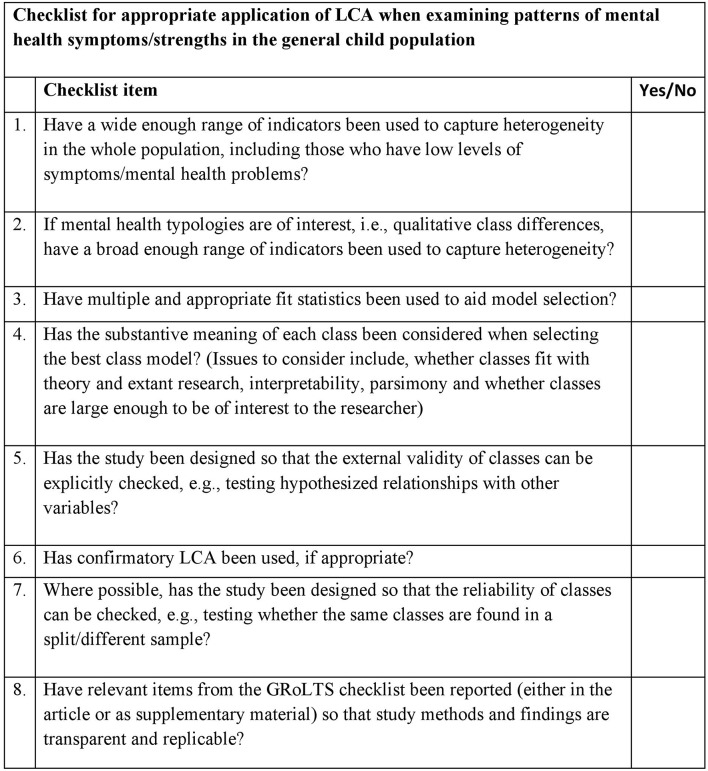
Checklist for appropriate application of LCA, when examining patterns of mental health symptoms/strengths in the general child population.

Despite those limitations, this is the first study to systematically review the application of LCA to investigate subgroups of children with similar mental health symptoms and strengths, in the general population. It has not only compared the results from studies in this area, but also indicated which different model selection methods have been applied and the extent to which studies evaluated evidence for class validity and reliability. In doing so, the review highlights that LCA has been used effectively in the field, although further work is needed to improve the rigor with which the method is applied. A checklist for practitioners or researchers intending to use LCA for the study of population mental health is provided (see Figure 2). This can be used to improve the application of LCA in this area in the future.

## Author Contributions

All authors contributed to the conception and design of the study. KP devised the search terms, searched the databases, did an initial sort based on paper titles and abstracts, and a further full paper sort of remaining papers, against the inclusion/exclusion criteria. PQ sorted over 20% of papers in the initial title and abstract sorting phase and 100% of papers in the full text sorting phase to ensure consistency in sorting. Any ambiguities in sorting were discussed among all authors. KP carried out data extraction and tabulated the results. KP wrote the manuscript and PQ and NH contributed to manuscript revision. All authors read, and approved the submitted version.

### Conflict of Interest Statement

The authors declare that the research was conducted in the absence of any commercial or financial relationships that could be construed as a potential conflict of interest.

## Footnotes

^1^Data extraction sheet template available as Supplementary Material S2.

^2^Adapted GRoLTS checklist available as Supplementary Material S3.
