# Supplementary material for: The Application of Latent Class Analysis for Investigating Population Child Mental Health: A Systematic Review
Source: Front Psychol. 2019 May 29;10:1214. doi: 10.3389/fpsyg.2019.01214 (PMC6548989; doi:10.3389/fpsyg.2019.01214)
Supplement: Supplementary file 1 [file Table_1.DOCX]

**Supplementary material (S1)**

***Table of search terms and filters used for each database***

| **Database** | **Search terms** | **Filters** |
| --- | --- | --- |
| PsycInfo (1806 to December Week 4 2017) | child*.mp. or elementary schools/  AND  (person cent?red or person oriented or person orientated or latent class or LCA or latent profile or LPA or mixture model*).mp.  AND  mental health/ or well being/ or exp CHILD PSYCHOPATHOLOGY/ or exp Behavior Disorders/ or exp Emotions/ or exp Affective Disorders/ or exp Emotional Disturbances/ or exp Mental Disorders/ or exp Anxiety Disorders/ or exp Anxiety/ or exp "DEPRESSION (EMOTION)"/ or loneliness.mp. or exp Behavior Problems/ or exp Attention Deficit Disorder with hyperactivity/ or exp CONDUCT DISORDER/ or exp Aggressive Behavior/ or exp Hyperkinesis/ or *"quality of life"/ or exp Life Satisfaction/ or exp Emotional States/ or exp Happiness/ or exp EMOTIONS/ or *peer relations/ or *antisocial behavior/ | English language only, 1997-current |
| MEDLINE (1946-december week 4 2017) | (Child Behaviour Disorders/ or Child Psychiatry/ or Psychology, Child/ or Child Behavior/ or Child/ or Child Development/ or Child Welfare/) and (person cent?red analys* or person oriented analys* or person orientated analys* or latent class* or LCA or latent profile* or LPA or mixture model*).mp. and (mental competency/ or mental health/ or mental processes/ or personal autonomy/ or resilience, psychological/ or mental disorders/ or internali#ing.mp. or externali#ing.mp. or anxiety disorders/ or mood disorders/ or "attention deficit and disruptive behavior disorders"/ or attention deficit disorder with hyperactivity/ or conduct disorder/ or child behavior disorders/ or "Disruptive, Impulse Control, and Conduct Disorders"/ or Depression/ or Anxiety/ or Loneliness/ or Aggression/ or "Quality of Life"/ or exp Affect/ or Psychopathology/ or wellbeing.mp. or exp Emotions/ or antisocial behavio?r.mp. or *Interpersonal Relations/) [mp=title, abstract, original title, name of substance word, subject heading word, keyword heading word, protocol supplementary concept word, rare disease supplementary concept word, unique identifier, synonyms] | English language only, 1997-current |
|  |  |  |
|  |  |  |
| Supplementary Table 1 (continued) | | |
| Database | Search terms | Filters |
| ASSIA (2001-2017) | ((child* OR MAINSUBJECT.EXACT.EXPLODE("Primary schools")) AND (MAINSUBJECT.EXACT.EXPLODE("Person centred") OR MAINSUBJECT.EXACT.EXPLODE("Person centred approach") OR MAINSUBJECT.EXACT.EXPLODE("Latent class analysis") OR ("latent profile*" OR LCA OR LPA) OR ("mixture model*"))) AND (MAINSUBJECT.EXACT.EXPLODE("Mental health") OR MAINSUBJECT.EXACT.EXPLODE("Internalizing problems") OR MAINSUBJECT.EXACT.EXPLODE("Internalizing behaviour") OR MAINSUBJECT.EXACT.EXPLODE("Externalizing behaviour") OR MAINSUBJECT.EXACT.EXPLODE("Externalizing problems") OR MAINSUBJECT.EXACT.EXPLODE("Childhood depression") OR MAINSUBJECT.EXACT.EXPLODE("Anxiety-Depression") OR MAINSUBJECT.EXACT.EXPLODE("Anxiety") OR MAINSUBJECT.EXACT.EXPLODE("Anxiety disorders") OR MAINSUBJECT.EXACT.EXPLODE("Life satisfaction") OR MAINSUBJECT.EXACT.EXPLODE("Emotional wellbeing") OR MAINSUBJECT.EXACT.EXPLODE("Spiritual wellbeing") OR MAINSUBJECT.EXACT.EXPLODE("Psychosocial wellbeing") OR MAINSUBJECT.EXACT("Wellbeing") OR MAINSUBJECT.EXACT.EXPLODE("Subjective wellbeing") OR MAINSUBJECT.EXACT.EXPLODE("Psychological wellbeing") OR MAINSUBJECT.EXACT.EXPLODE("Loneliness") OR MAINSUBJECT.EXACT.EXPLODE("Worry") OR MAINSUBJECT.EXACT.EXPLODE("Attention deficit hyperactivity disorder") OR MAINSUBJECT.EXACT.EXPLODE("Hyperactivity") OR MAINSUBJECT.EXACT.EXPLODE("Aggression") OR MAINSUBJECT.EXACT.EXPLODE("Conduct") OR MAINSUBJECT.EXACT.EXPLODE("Conduct disordered children") OR MAINSUBJECT.EXACT.EXPLODE("Affective disorders") OR MAINSUBJECT.EXACT.EXPLODE("Affective experiences") OR MAINSUBJECT.EXACT.EXPLODE("Positive affect") OR MAINSUBJECT.EXACT.EXPLODE("Negative affect") OR MAINSUBJECT.EXACT.EXPLODE("Affective symptoms") OR MAINSUBJECT.EXACT.EXPLODE("Affect") OR MAINSUBJECT.EXACT.EXPLODE("Affective responses") OR MAINSUBJECT.EXACT("Quality of life") OR MAINSUBJECT.EXACT("Interpersonal relationships") OR MAINSUBJECT.EXACT("Psychopathology") OR MAINSUBJECT.EXACT("Antisocial behaviour") OR MAINSUBJECT.EXACT.EXPLODE("Emotions")) | English language only;  Date range 2001-2017 |
|  |  |  |
| Supplementary Table 1 (continued) | | |
| Database | Search terms | Filters |
| EMBASE (1980-2007 week 4) | (child*.mp. or primary school/) and (person cent?red or person oriented or person orientated or latent class* or LCA or latent profile* or LPA or mixture model*).mp. and (mental health/ or *wellbeing/ or externali#ing.mp. or internali#ing.mp. or emotional disorder/ or behavior disorder/ or problem behavior/ or child behavior/ or disruptive behavior/ or *mood disorder/ or *depression/ or *anxiety/ or *fear/ or loneliness/ or *aggression/ or *aggressiveness/ or *anger/ or hyperactivity/ or life satisfaction/ or affect/ or exp peer group/ or antisocial behavior/ or exp emotion/ or *"quality of life"/) | English language only, 1997-current |
| ERIC | (child* OR MAINSUBJECT.EXACT.EXPLODE("Elementary Schools")) AND ("person oriented" OR "person orientated" OR "person centered" OR "person centred" OR "latent class*" OR LCA OR "latent profile*" OR LPA OR "mixture model*") AND (MAINSUBJECT.EXACT.EXPLODE("Mental Health") OR MAINSUBJECT.EXACT.EXPLODE("Well Being") OR MAINSUBJECT.EXACT("Behavior Patterns") OR MAINSUBJECT.EXACT("Behavior") OR MAINSUBJECT.EXACT("Psychiatry") OR MAINSUBJECT.EXACT("Psychopathology") OR MAINSUBJECT.EXACT("Antisocial Behavior") OR MAINSUBJECT.EXACT("Mental Disorders") OR MAINSUBJECT.EXACT("Emotional Disturbances") OR MAINSUBJECT.EXACT("Attention Deficit Disorders") OR MAINSUBJECT.EXACT.EXPLODE("Behavior Disorders") OR MAINSUBJECT.EXACT("Behavior Problems") OR externali?ing OR internali?ing OR MAINSUBJECT.EXACT("Depression (Psychology)") OR MAINSUBJECT.EXACT("Fear") OR MAINSUBJECT.EXACT("Anxiety") OR MAINSUBJECT.EXACT("Helplessness") OR MAINSUBJECT.EXACT("Emotional Disturbances") OR MAINSUBJECT.EXACT("Emotional Adjustment") OR MAINSUBJECT.EXACT("Emotional Problems") OR MAINSUBJECT.EXACT("Psychological Patterns") OR MAINSUBJECT.EXACT.EXPLODE("Aggression") OR MAINSUBJECT.EXACT.EXPLODE("Hyperactivity") OR MAINSUBJECT.EXACT.EXPLODE("Attention Deficit Hyperactivity Disorder") OR MAINSUBJECT.EXACT("Quality of Life") OR MAINSUBJECT.EXACT.EXPLODE("Life Satisfaction") OR ("positive affect" OR "negative affect") OR MAINSUBJECT.EXACT.EXPLODE("Peer Relationship") OR MAINSUBJECT.EXACT.EXPLODE("Antisocial Behavior")) | English language only and date 1/1/1997 to 31/12/2017 |
|  |  |  |
|  | | |
| Supplementary Table 1 (continued) | | |
| Database | Search terms | Filters |
| Scopus | ( TITLE-ABS ( child*  OR  "primary school"  OR  "elementary school" ) )  AND  ( TITLE-ABS ( "person centred"  OR  "person oriented"  OR  "latent class*"  OR  lca  OR  "latent profile*"  OR  lpa  OR  "mixture model" ) )  AND  ( TITLE-ABS ( "mental health"  OR  "well being"  OR internali?ing  OR  externali?ing  OR  "emotional problem*"  OR  psychopathology  OR  "emotional disorder*"  OR  "emotional difficult*"  OR  "behavio* problem*"  OR  "behavio* difficult*"  OR  "behavio* disorder*"  OR  depressi*  OR  anxiety  OR  loneliness  OR  worry  OR conduct  OR  aggressi*  OR  hyperactiv*  OR  "life-satisfaction"  OR  "positive affect"  OR  "negative affect"  OR  "affective state"  OR  happiness  OR  {quality of life}  OR  "antisocial behavio*"  OR  "peer problems" ) ) | Limit to English Language and year 1997-2017 |
| Google Scholar | child + "person oriented \| centred" \| "latent class \| profile " \| "mixture model" + "mental health" \| "well being" \| psychopathology \| life-satisfaction \| externalising \| internalising \| conduct \| depression \| anxiety \| aggression \| loneliness \| happiness | date limit 1997-2017 English Language only |
