# Supplementary material for: The Application of Latent Class Analysis for Investigating Population Child Mental Health: A Systematic Review
Source: Front Psychol. 2019 May 29;10:1214. doi: 10.3389/fpsyg.2019.01214 (PMC6548989; doi:10.3389/fpsyg.2019.01214)
Supplement: Supplementary file 2 [file Table_2.DOCX]

**Supplementary material (S2)**

***Data extraction sheet template***

| Reference information | Authors |
| --- | --- |
|  | Year |
|  | Title |
|  | Journal |
|  | Reference |
| Paper aims/scope | Reason for using LCA |
|  | Hypotheses/predictions |
|  | Exploratory or confirmatory? |
|  | Psychopathology/subjective wellbeing/both |
|  | Broad/narrow dimension of mental health investigated? |
| Sample | Participant age |
|  | Participant gender |
|  | Participant ethnicity |
|  | Country |
|  | Sample size |
|  | Dataset |
|  | Other notes on representativeness |
| Variables and measures | Mental health indicators |
|  | Number of indicators used in LCA |
|  | Measures used for mental health indicators |
|  | Covariates/distal outcomes (if any) and measure |
|  | Reporter |
|  | Cross sectional or longitudinal? |
| Analysis | LPA or LCA |
|  | Software used |
|  | Fit statistics used |
|  | Theoretical justification used to determine groups? |
|  | Number of models fit |
|  | Method for including covariates |
|  | Other analyses, e.g. grouping variables, crosstabs |
| Results | Final number of classes |
|  | Class names and prevalences |
|  | Reliability of classes tested in other or split sample? |
|  | Covariates which predicted classes (antecedents) |
|  | Covariates which were predicted by class (outcomes) |
|  | Covariates in cross-sectional studies |
|  | Grouping variable effects |
|  | Other analyses |
| Items on the adapted GRoLTS checklist | Is the missing data mechanism reported? |
|  | Is a description provided of what variables are related to attrition or missing data? |
|  | Is a description provided of how missing data in the analysis was dealt with? |
|  | Is information about the distribution of the observed variables included? |
|  | Is the software mentioned? |
|  | Are parameter restrictions reported? |
|  | If covariates have been used, can analyses still be replicated? i.e., is sufficient detail provided about the method used to include covariates? |
|  | Is information reported about the number of random start values and final iterations included? |
|  | Are the model comparison (and selection) tools described from a statistical perspective? i.e., transparent about how they selected the model using which indices |
|  | Are the total number of fitted models reported, including a one-class solution? |
|  | Are the number of cases per class reported for each model? |
|  | Is entropy reported? |
|  | Are plots/bar charts included with the response patterns of the classes/profiles in the final solution? |
|  | Are plots/bar charts included with the response patterns of the classes/profiles for each model? |
|  | Are characteristics of the final class solution numerically described? |
|  | Are the syntax files available? |
