# Supplementary material for: The Application of Latent Class Analysis for Investigating Population Child Mental Health: A Systematic Review
Source: Front Psychol. 2019 May 29;10:1214. doi: 10.3389/fpsyg.2019.01214 (PMC6548989; doi:10.3389/fpsyg.2019.01214)
Supplement: Supplementary file 3 [file Table_3.DOCX]

**Supplementary material (S3)**

***Adaption of the Guidelines for Reporting on Latent Trajectory Studies (GRoLTS) checklist for LCA studies***

| **GRoLTS checklist (van de Schoot et al., 2017)** | **Adaption for LCA studies** |
| --- | --- |
| 1. Is the metric of time used in the statistical model reported? | Item not applicable for LCA/LPA |
| 2. Is information presented about the mean and variance of time within a wave? | Item not applicable for LCA/LPA |
| 3a. Is the missing data mechanism reported? | Is the missing data mechanism reported? |
| 3b. Is a description provided of what variables are related to attrition/missing data? | Is a description provided of what variables are related to attrition/ missing data? |
| 3c. Is a description provided of how missing data in the analyses were dealt with? | Is a description provided of how missing data in the analyses were dealt with? |
| 4. Is information about the distribution of the observed variables included? | Is information about the distribution of the observed variables included? e.g., continuous, ordinal, count, zero inflated, |
| 5. Is the software mentioned? | Is the software mentioned? |
| 6a. Are alternative specifications of within-class heterogeneity considered (e.g., LGCA vs. LGMM) and clearly documented? If not, was sufficient justification provided as to eliminate certain specifications from consideration? | Are parameter restrictions reported? |
| 6b. Are alternative specifications of the between-class differences in variance–covariance matrix structure considered and clearly documented? If not, was sufficient justification provided as to eliminate certain specifications from consideration? | Item not applicable for LCA/LPA |
| 7. Are alternative shape/functional forms of the trajectories described? | Item not applicable for LCA/LPA |
| 8. If covariates have been used, can analyses still be replicated? | If covariates have been used, can analyses still be replicated? i.e., is sufficient detail provided about the method used to include covariates? |
| 9. Is information reported about the number of random start values and final iterations included? | Is information reported about the number of random start values and final iterations included? |
| 10. Are the model comparison (and selection) tools described from a statistical perspective? | Are the model comparison (and selection) tools described from a statistical perspective? |
| 11. Are the total number of fitted models reported, including a one-class solution? | Are the total number of fitted models reported, including a one-class solution? |
| 12. Are the number of cases per class reported for each model (absolute sample size, or proportion)? | Are the number of cases per class reported for each model (absolute sample size, or proportion)? |
| 13. If classification of cases in a trajectory is the goal, is entropy reported? | Is entropy reported? |
| 14a. Is a plot included with the estimated mean trajectories of the final solution? | Are plots/barcharts included with the response patterns of the classes/profiles in the final solution? |
| 14b. Are plots included with the estimated mean trajectories for each model? | Are plots/barcharts included with the response patterns of the classes/profiles for each model? |
| 14c. Is a plot included of the combination of estimated means of the final model and the observed individual trajectories split out for each latent class? | Item not applicable for LCA/LPA |
| 15. Are characteristics of the final class solution numerically described (i.e., means, SD/SE, n, CI, etc.)? | Are characteristics of the final class solution numerically described? (i.e., means, SD/SE, n, CI, etc.) |
| 16. Are the syntax files available (either in the appendix, supplementary materials, or from the authors)? | Are the syntax files available (either in the appendix, supplementary materials, or from the authors)? |
