# Supplementary material for: The Application of Latent Class Analysis for Investigating Population Child Mental Health: A Systematic Review
Source: Front Psychol. 2019 May 29;10:1214. doi: 10.3389/fpsyg.2019.01214 (PMC6548989; doi:10.3389/fpsyg.2019.01214)
Supplement: Supplementary file 4 [file Table_4.DOCX]

| **Supplementary material (S4)**  ***Table showing quality assessment results based on items from the Guidelines for Reporting on Latent Trajectory Studies (GRoLTS) checklist*** | | | | | | | | | | | | | | | | |
| --- | --- | --- | --- | --- | --- | --- | --- | --- | --- | --- | --- | --- | --- | --- | --- | --- |
|  | Reports missing data mechanism | Describes variables related to missing data | Describes how missing data dealt with | Distribution of the observed variables reported | Software mentioned | Parameter restrictions reported | Covariate analyses can be replicated | Random start values and final iterations reported | Model selection tools described statistically | Number of fitted models reported, including 1-class | Number of cases per class reported for each model | Entropy reported | Plots/bar charts included for the final solution | Plots/bar charts included for each model | Final class solution numerically described | Syntax files available |
| **Althoff et al. (2006)** |  |  | * | * | * |  | n/a |  | * | * |  |  | * |  | * |  |
| **Althoff et al. (2009)** |  |  |  |  | * |  |  |  | * | * |  |  | * |  | * |  |
| **Baillargeon et al. (1999)** | * |  | * |  | * | * | n/a |  | * | * |  |  | * |  | * |  |
| **Basten et al. (2013)** |  | * | * |  | * |  | * |  | * | * |  | * | * |  | * |  |
| **Basten et al. (2016)** |  | * | * |  | * |  | n/a |  | * | * |  | * | * |  | * |  |
| **Bradshaw et al. (2015)** | * | * | * |  | * |  | * |  | * | * | * | * | * |  | * |  |
| **Carey et al. (2017)** |  |  | * | * | * | * | * |  | * |  |  | * | * |  | * |  |
| **Ferdinand et al. (2006)** |  |  |  |  | * | * | n/a |  | * | * |  | * | * |  | * |  |
| **Fergusson et al. (2009)** |  | * | * | * | * |  | * |  | * |  |  |  |  |  | * |  |
| **Hudziak et al. (1999)** |  |  |  | * | * |  | n/a |  | * | * |  | * | * |  | * |  |
| **Kuny et al. (2013)** |  |  |  |  | * | * | * |  | * | * |  | * | * |  | * |  |
| **H. I. Lanza (2011)** |  |  | * |  | * |  | * |  | * | * |  |  |  |  | * |  |
| **Lee et al. (2007)** |  |  | * |  | * | * | n/a |  | * | * |  |  |  |  | * |  |
| **McElroy et al. (2017)** |  |  |  |  |  |  | n/a | * | * | * |  | * | * |  | * |  |
| **Nozadi et al. (2016)** | * | * | * | * | * |  | * |  | * | * |  | * | * |  | * |  |
| **Racz et al. (2015)** |  | * | * | * | * |  | n/a |  | * | * | * | * | * |  | * |  |
| **Romano et al. (2002)** |  |  |  | * | * | * | n/a |  | * | * |  |  |  |  | * |  |
| **Sulik et al. (2017)** |  |  |  |  | * | * | * |  | * |  |  | * | * |  | * |  |
|  | | | | | | | | | | | | | | | | |
| **Supplementary Table 2 (continued)** | | | | | | | | | | | | | | | | |
|  | Reports missing data mechanism | Describes variables related to missing data | Describes how missing data dealt with | Distribution of the observed variables reported | Software mentioned | Parameter restrictions reported | Covariate analyses can be replicated | Random start values and final iterations reported | Model selection tools described statistically | Number of fitted models reported, including 1-class | Number of cases per class reported for each model | Entropy reported | Plots/bar charts included for the final solution | Plots/bar charts included for each model | Final class solution numerically described | Syntax files available |
| **van Lier, Verhulst, van der Ende, et al. (2003)** |  |  |  |  | * |  | * |  | * |  |  |  | * |  | * |  |
| **van Lier, Verhulst, & Crijnen (2003)** |  | * |  |  | * |  | * |  | * |  |  |  | * |  | * |  |
| **Vendlinski et al., (2014)** |  |  |  |  | * |  | * | * | * | * |  |  | * |  | * |  |
| **Wadsworth et al. (2001)** |  |  |  |  | * |  | n/a |  |  | * |  |  | * |  | * |  |
| **Wall et al. (2016)** |  |  |  |  | * |  |  |  | * |  |  | * | * |  | * |  |
|  |  |  |  |  |  |  |  |  |  |  |  |  |  |  |  |  |
| Total yes | 3 | 7 | 12 | 7 | 22 | 7 | 11 | 2 | 22 | 17 | 2 | 12 | 19 | 0 | 23 | 0 |
| Total no | 20 | 16 | 11 | 16 | 1 | 16 | 2 | 21 | 1 | 6 | 21 | 11 | 4 | 23 | 0 | 23 |
| **% yes** | **13** | **30** | **52** | **30** | **96** | **30** | **85** | **9** | **96** | **74** | **9** | **52** | **83** | **0** | **100** | **0** |
| *GRoLTS results* (van de Schoot et al., 2017) | *5* | *61* | *87* | *18* | *95* | *29* | *86* | *3* | *97* | *63* | *16* | *93* | *100* | *0* | *63* | *5* |
